# Multiple congenital visceral abnormalities as a rare cause of pulmonary arterial hypertension

**DOI:** 10.1186/s43044-022-00273-x

**Published:** 2022-04-28

**Authors:** Parham Rabiee, Sedigheh Saedi

**Affiliations:** 1 Radiology Department, Rajaei Cardiovascular Medical and Research Center, Iran University of Medical Sciences, Tehran, Iran; 2 Cardiology and Adult Congenital Heart Disease Department, Rajaei Cardiovascular Medical and Research Center, Iran University of Medical Sciences, Vali-asr Ave, Adjacent to Mellat Park, 1995614331 Tehran, Iran

**Keywords:** Congenital heart disease, Pulmonary hypertension, Mayer–Rokitansky–Küster–Hauser syndrome, Abernethy malformation

## Abstract

**Background:**

Pulmonary arterial hypertension (PAH) is a rare, progressive disorder. PAH is caused by a wide spectrum of pathologies but the cause remains undetermined on many occasions and patients are classified in the idiopathic group.

**Case presentation:**

Here we report a young woman with rare congenital visceral abnormalities presenting with severe pulmonary hypertension.

**Conclusions:**

Pulmonary hypertension is a complex disorder. Search for uncommon conditions that lead to pulmonary hypertension is necessary to determine the best management options.

**Supplementary Information:**

The online version contains supplementary material available at 10.1186/s43044-022-00273-x.

## Background

Pulmonary hypertension (PH) is defined as an increase in mean pulmonary arterial pressure of 20 mm Hg or greater at rest with the stipulation that pulmonary vascular resistance (PVR) is ≥ 3 Woods units, as evaluated by right heart catheterization. In PH there is pathologic medial hypertrophy, intimal proliferation and fibrotic changes mainly in the distal pulmonary arteries. Remodeling in the pulmonary vasculature leads to right heart failure and progressive decline in functional capacity. The early symptoms of PH are non-specific, and the diagnosis is often made in advanced stages of the disease with worse long-term outcomes [1]. PH associated with portal hypertension or portopulmonary hypertension is an important cause of increased pulmonary arterial pressure with or without chronic liver disease. Portopulmonary hypertension is currently classified in group 1 of pulmonary hypertension classification [2, 3]. Here we report a young woman with previously undetected complex congenital visceral abnormalities presenting with severe pulmonary hypertension.

## Case presentation

An 18-year-old asymptomatic female who was candidate for vaginal reconstruction surgery due to Mayer–Rokitansky–Küster–Hauser (MRKH) syndrome was referred to our tertiary care center following incidental finding of right sided cardiac chamber enlargement and severe pulmonary arterial hypertension in a transthoracic echocardiography performed prior to the gynecologic surgery. The patient had uterine agenesis and primary amenorrhea with normal external genitalia. She showed normal developmental and pubertal milestones. On genetic study she had normal female karyotype of 46, XX. In physical examination there was scoliosis of the spinal bones.

**Fig. 1:**
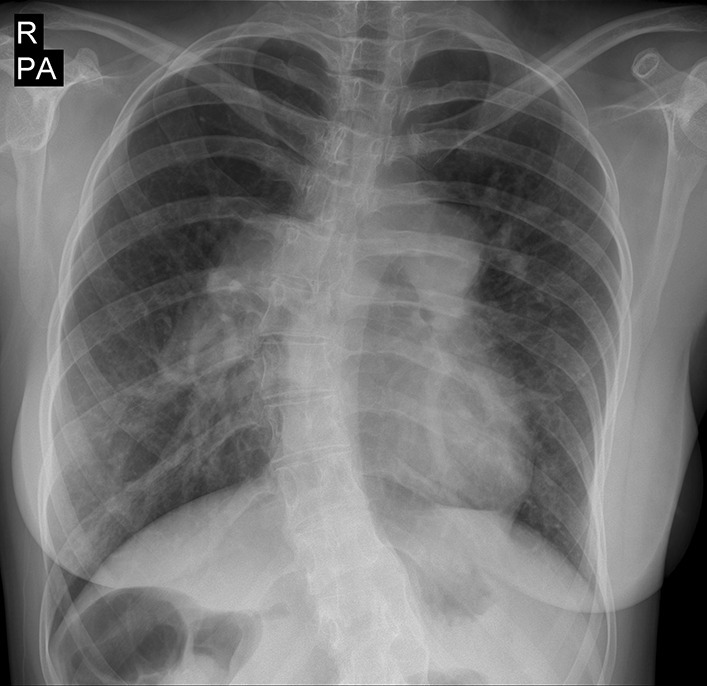
Chest X-ray showing pulmonary artery dilation, right ventricular enlargement and scoliosis

**Fig. 2:**
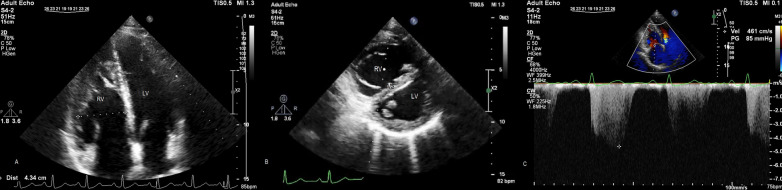
Echocardiogram showing right ventricular enlargement (**A**), septal flattening in systole and diastole in favor of right ventricular pressure overload (**B**), and high tricuspid regurgitation gradients (**C**). *RV* right ventricle, *LV* left ventricle, *IVS* inter-ventricular septum

Chest X-ray depicted dilated pulmonary artery, right ventricular enlargement and scoliosis (Fig. 1). In twelve lead ECG there was normal sinus rhythm and right axis deviation. Transthoracic echocardiography showed right ventricular dilation and estimated systolic pulmonary artery pressure of about 93 mmHg (Fig. 2). Transesophageal echocardiography was performed and showed no intra-cardiac shunt. There was also no bubble passage during contrast injection with and without provocative maneuvers.

Laboratory data were negative for autoimmune and rheumatic causes of pulmonary hypertension. We found a relatively low platelet count of 118,000 per microliter of blood plus mild increases in liver enzymes and total bilirubin levels. Although the patient had previously undergone abdomino-pelvic sonography for her gynecologic problems that only reported uterine agenesis, we decided to perform a repeat sonography in search for possible liver abnormalities or portal hypertension with regards to unexplained thrombocytopenia. Interestingly abdominal sonography showed that the portal vein was totally absent and right liver lobe was atrophic.

**Fig. 3:**
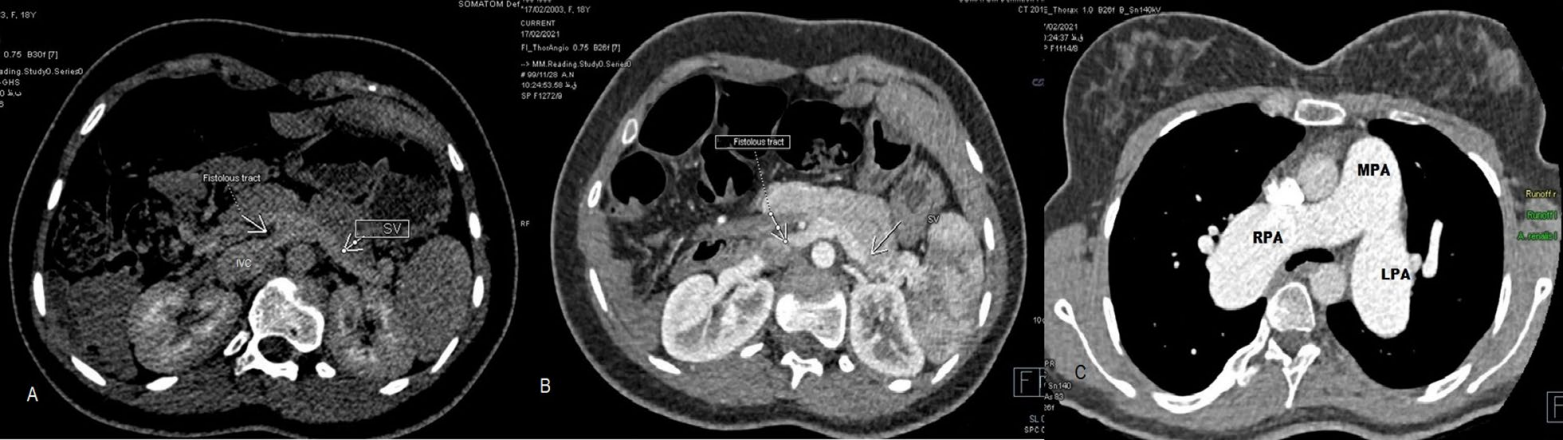
Contrast-enhanced axial CT scan in porto-venous phase demonstrating abnormal fistulous tract directly connecting splenic vein (SV) and superior mesenteric vein to inferior vena cava with absent main portal vein (**A**, **B**). Axial contrast CT image showing dilated pulmonary artery in favor of significant pulmonary hypertension (**C**)

Further evaluation by abdominal CT-angiography revealed congenital absence of intra-hepatic portal branches with direct drainage of abdominal veins to inferior vena cava resulting in extra hepatic porto-systemic shunt in favor of Abernathy type 1 visceral malformation. There was also gut malrotation and evidence of MRKH syndrome with underdeveloped uterus and normal ovaries. Computed tomographic angiography of the heart and pulmonary arteries showed no evidence of pulmonary parenchymal involvement and no chronic pulmonary thromboembolic disease or vascular shunts (Fig. 3).

**Fig. 4:**
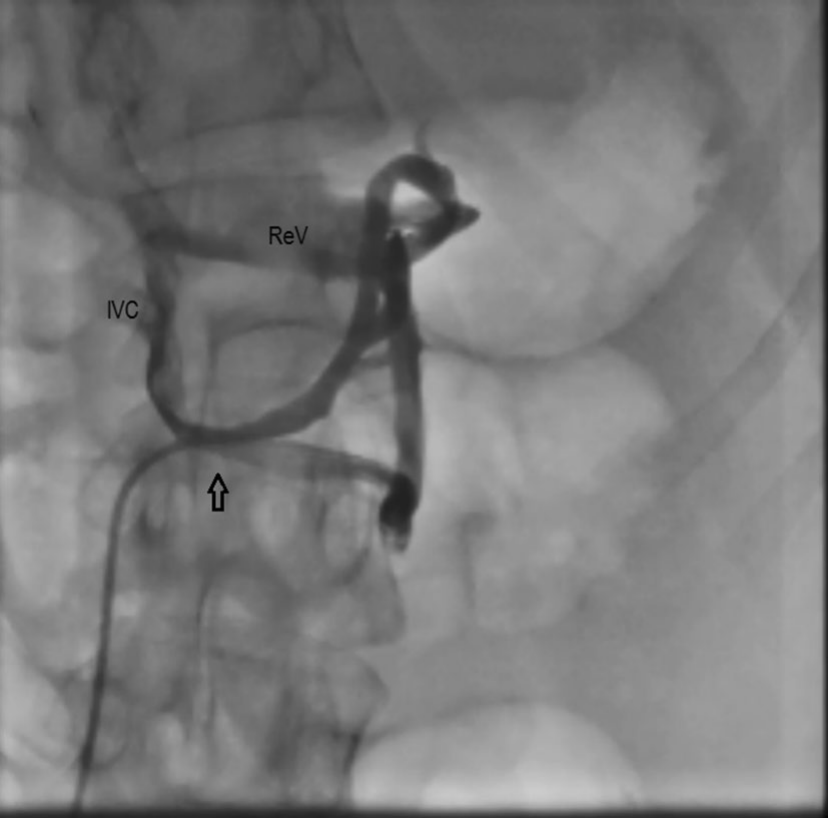
Abdominal angiography showing absent portal vein and splenic and superior mesenteric veins draining directly to inferior vena cava (arrow). *IVC* inferior vena cava, *ReV* renal vein

Right heart catheterization data confirmed severe pre-capillary pulmonary hypertension with pulmonary arterial pressure of 100/40 mmHg, normal cardiac output, normal capillary wedge pressure and no left to right shunts. Abdominal vascular angiography showed the complete absence of portal vein with splenic and superior mesenteric veins confluence draining directly to inferior vena cava forming a mesentero-systemic shunt (Fig. 4, Additional file 1: Video S1).

Unfortunately as the portal vein was entirely absent elimination of the porto-systemic shunt by interventional or surgical methods was not possible. The patient is a candidate for future lung and liver transplantation and was discharged home on pulmonary vasodilator therapy with sildenafil and macitentan.

## Discussion

Pulmonary hypertension often presents with nonspecific symptoms including exertional dyspnea, progressive decline in exercise capacity, angina or syncope. Patients are frequently detected incidentally during a transthoracic echocardiography performed for other indications. Echocardiography findings favoring remarkable PH warrant detailed clinical evaluation to establish the specific etiology. Comprehensive laboratory-tests, transesophageal echocardiography, pulmonary function tests, high-resolution CT, abdominal sonography and CT pulmonary angiography are undertaken based on the presumed underlying cause of PH. Cardiac magnetic resonance (CMR) imaging helps delineate cardiac morphology and function and non-invasively assess the hemodynamics. CMR also provides valuable prognostic information. Right heart catheterization is necessary to confirm the diagnosis, ascertain PH severity and carry out vasoreactivity testing when clinically indicated [3–5].

Pulmonary hypertension occurs in about 1–5% of patients with portal hypertension. Abernethy malformation or congenital extra hepatic portosystemic shunt is a rare abnormality of the portal venous system with shunting of portal blood into the systemic venous system. Abernethy malformation has two subtypes. In type I that is more frequent in females, the portal vein and intrahepatic portal circulation are entirely absent. In type II the portal vein is hypoplastic. In the absence of portal venous system the intestinal and splenic veins drain directly to the inferior vena cava, left renal vein or the left hepatic vein creating a porto-systemic shunt and varying degrees of portal hypertension. As the normal enterohepatic circulation is diminished, the toxins and vasoactive material from the intestine are bypassed to the systemic circulation without being metabolized in the liver leading to liver damage, hepatopulmonary syndrome, pulmonary hypertension and arterio-venous fistulas. Pulmonary arterial hypertension develops as a result of chronic vasoconstrictive effect of toxic vasoactive substances on the pulmonary vascular bed. There is also an increased risk of hepatic neoplasms. Type II could be treated by surgery or interventional methods of shunt elimination [6–8]. Our patient had type I malformation in which the treatment option is limited to liver transplantation; however, presence of severe pulmonary hypertension serves as an obstacle to transplantation unless concurrent lung transplantation is performed.

Skeletal and visceral anomalies including hepatobilliary, splenic and genitourinary anomalies have been reported in Abernethy malformation [9]. Our patient had concomitant MRKH syndrome that is characterized by the failure of normal development of the uterus and the vagina. The ovarian function, external genitalia and development of secondary sexual characteristics during puberty are normal. Affected women do not have menstrual cycles (primary amenorrhea). MRKH syndrome could be isolated (Type I) or be associated with other defects including renal, skeletal, cardiac or hearing abnormalities (Type II). This syndrome has significant psychological effects. Management options include surgical or nonsurgical creation of a neovagnia or newly introduced methods of uterine transplantation [10]. Although associated cardiac defects are reported in MRKH type II patients but to the best of our knowledge co-occurrence with porto-systemic shunt and pulmonary hypertension has not been reported previously. Presence of multiple anomalies would further complicate treatment options in these patients.

## Conclusions

Pulmonary hypertension is a complicated and multifactorial disorder requiring multidisciplinary assessment. Search for unusual and rare conditions that may cause pulmonary hypertension including congenital visceral abnormalities is important in determining the prognosis and treatment.

## Supplementary Information

**Additional file 1:** Abdominal vascular angiography showing  absence of portal vein with splenic and superior mesenteric veins confluence draining directly to inferior vena cava.

## Data Availability

Presented in the main paper.

## Abbreviations

PH: Pulmonary hypertension
RV: Right ventricle
MRKH: Mayer–Rokitansky–Küster–Hauser
